# Intradermal delivery of STAT3 siRNA to treat melanoma via dissolving microneedles

**DOI:** 10.1038/s41598-018-19463-2

**Published:** 2018-01-18

**Authors:** Jingtong Pan, Wenyi Ruan, Mengyao Qin, Yueming Long, Tao Wan, Kaiyue Yu, Yuanhao Zhai, Chuanbin Wu, Yuehong Xu

**Affiliations:** School of Pharmaceutical Sciences, Sun Yat-sen University, Guangzhou, 510006 China

## Abstract

Hyperactivity of signal transducer and activity of transcription 3 (STAT3) plays a crucial role in melanoma invasion and metastasis. Gene therapy applying siRNA targeting STAT3 is a potential therapeutic strategy for melanoma. In this article, we first fabricated safe and novel dissolving microneedles (MNs) for topical application of STAT3 siRNA to enhance the skin penetration of siRNA and used polyethylenimine (PEI, 25 kDa) as carrier to improve cellular uptake of siRNA. The results showed that MNs can effectively penetrate skin and rapidly dissolve in the skin. *In vitro* B16F10 cell experiments presented that STAT3 siRNA PEI complex can enhance cellular uptake and transfection of siRNA, correspondingly enhance gene silencing efficiency and inhibit tumor cells growth. *In vivo* experiments indicated that topical application of STAT3 siRNA PEI complex delivered by dissolving MNs into skin can effectively suppress the development of melanoma through silencing STAT3 gene, and the inhibition effect is dose-dependent. STAT3 siRNA delivery via dissolving MNs is a promising approach for skin melanoma treatment with targeting inhibition efficacy and minimal adverse effects.

## Introduction

Melanoma, a severe malignant tumor, initiates in melanocytes^1^. It leads to ~75% deaths associated with skin cancer^2^. In recent years, the incidence of melanoma is dramatically increasing^3^. Melanoma is hard to cure and causes high mortality in the clinic due to poor prognosis, aggressive local growth, high rate of metastasis, and resistance to traditional radiotherapy^4^. The typical therapeutic options for melanoma include surgical resection, chemotherapy, immunotherapy, radiotherapy, and biotherapy^5^. However, the therapeutic efficacy of these approaches is restricted due to drug resistance, high toxicity, and poor selectivity^1,6–8^. Small interfering RNA (siRNA) therapy is a promising gene therapy for tumor with relative low toxicity and high specificity^1,6–9^. In most malignant tumors, signal transducer and activator of transcription 3 (STAT3) is related to malignant behaviors including tumor proliferation, metastasis, angiogenesis, survival, and immune evasion^10^, and it is hyperactive in many malignant cancers such as breast cancer, prostate cancer, and melanoma^11–13^. The activation of STAT3 can increase the code of apoptosis inhibition genes Bcl-xl, Bcl-2, Mcl-1, and survivin^14^, and improve the expression of cell cycle regulatory cyclin D1 and myc^15,16^. The tumor cell apoptosis is consequently inhibited. Moreover, activated STAT3 in melanoma can directly combine to matrix metalloproteinase-2 (MMP-2) gene promoter to increase the expression of MMP-2. MMP-2 degrades extracellular matrix, thus promoting the invasion and metastasis of tumor cells^15–17^. Finally, the activated STAT3 improves the expression of VEGF in melanoma to stimulate angiogenesis of tumor and enhance the tumor vascular permeability^17–20^. Overall, sustained abnormal activation of STAT3 can inhibit tumor cell apoptosis, promote tumor cell proliferation, and enhance angiogenesis^21,22^. Some studies on melanoma have shown that STAT3 increases the expression of antiapoptotic genes Bcl-xl, Mcl-1 and further promote melanoma proliferation and malignant transformation^17^. Moreover, hyperactivity of STAT3 in melanoma increases the expression of VEGF to induce angiogenesis^23,24^. Down-regulation of STAT3 can inhibit the melanoma development and metastasis^24^. Hence, STAT3 is a potential target for melanoma therapy^12,25^.

Currently, the application of siRNA to treat skin diseases such as psoriasis, melanoma tumors, and atopic dermatitis attracts serious attentions from scientists^1,26^. However, delivery of macromolecular siRNA into skin remains a challenge. Stratum corneum (SC), the outermost layer of skin, consists of dead cells (corneocytes) imbedded in lamellar extracellular lipid matrix, which presents a significant barrier to the delivery of exogenous agents^27^. siRNAs are hydrophilic with high molecular weight (~13 kDa) and negative charge. These properties hinder siRNAs from penetrating SC. Melanocytes exist in the basal epidermis and upper dermis of skin^26,28^. Hence, siRNAs require to be delivered not only across SC but also through viable epidermis with the aim to reach the target of melanocytes. Microneedles (MNs) have been shown to overcome SC barrier, penetrate the skin and into the viable epidermis or dermis in a minimally invasive manner^29^, and they have been used for the delivery of macromolecules including human growth hormone^30^, interferon-α-2b^31^, hepatitis B surface antigen^32^, insulin^33–35^, anti-PD1 antibody^36^, and siRNA^37,38^. Therefore, we hypothesized that STAT3 siRNA could effectively be delivered via MNs to the deep layer of skin, readily accumulate in the local tumor, targetedly silence STAT3 gene, and directly inhibit the melanoma development without leakage into the systemic circulation for reducing the toxicity.

With the aim to test the hypothesis of topical siRNA targeting STAT3 as a gene therapy strategy for melanoma, we developed for the first time a novel intradermal delivery system for STAT3 siRNA based on dissolving MNs. The dissolving MNs were fabricated with biocompatible vehicles of hyaluronic acid (HA), dextran, and polyvinylpyrrolidone (PVP) 17. STAT3 siRNA was encapsulated with polymeric carrier polyethylenimine (PEI, 25KDa), and then loaded within dissolving MNs. The cellular uptake, anti-proliferation, and gene silencing efficiency of STAT3 siRNA PEI complex (PEI/siRNA) were observed with B16F10 melanoma cells *in vitro*, and the anti-melanoma activity of PEI/siRNA delivered by dissolving MNs was evaluated with mouse melanoma *in vivo*.

## Results

### Preparation and characteristics of dissolving MNs

**Figure 1:**
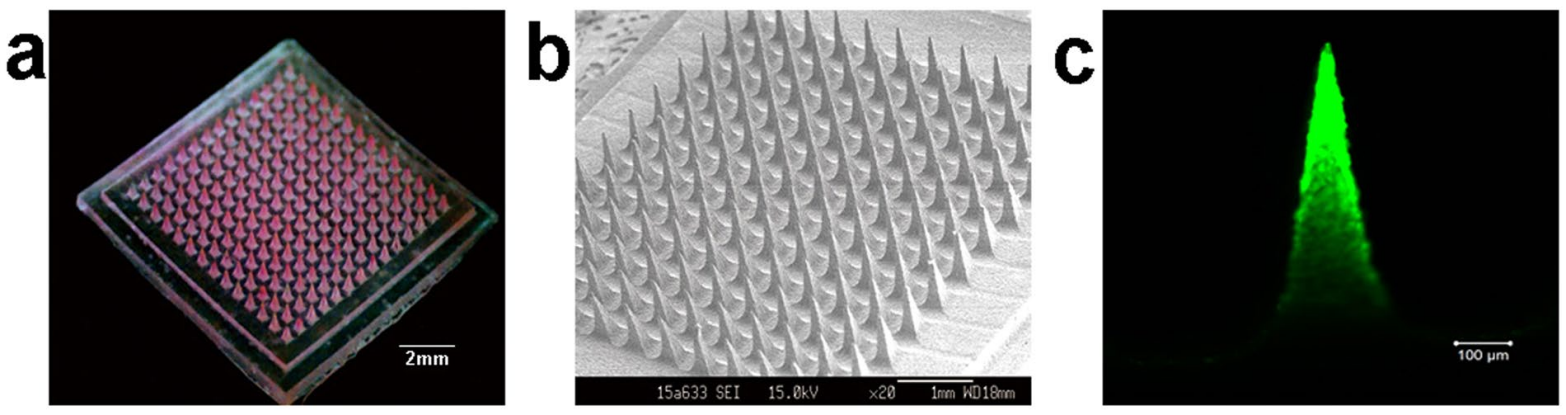
Images of dissolving microneedles. (**a**) The dissolving microneedle arrays loaded with Rhodamine B were photographed by digital camera. (**b**) The dissolving microneedle arrays were observed by scanning electron microscope. (**c**) The image of microneedle loaded with PEI/FAM-siRNA photographed by confocal laser scanning microscope.

The dissolving MNs were characterized in Fig. 1. Figure 1a presented the dissolving MNs loaded with Rhodamine B (Rh-B) photographed by digital camera with an area of 1 cm^2^. When the MNs patch was observed under scanning electron microscope, the MNs presented the arrays comprised of 144 (12 $\times 12$) needles with pyramidal shape (Fig. 1b). The needle was 650 μm in height, 300 μm in base, and 20 μm in the tip radius. The interspace between the arrays was 300 μm. After loaded with PEI/FAM-siRNA, the siRNA preferentially distributed in the upper tip of needle observed by confocal laser scanning microscope (CLSM, Fig. 1c).

### Mechanical strength and skin insertion studies

**Figure 2:**
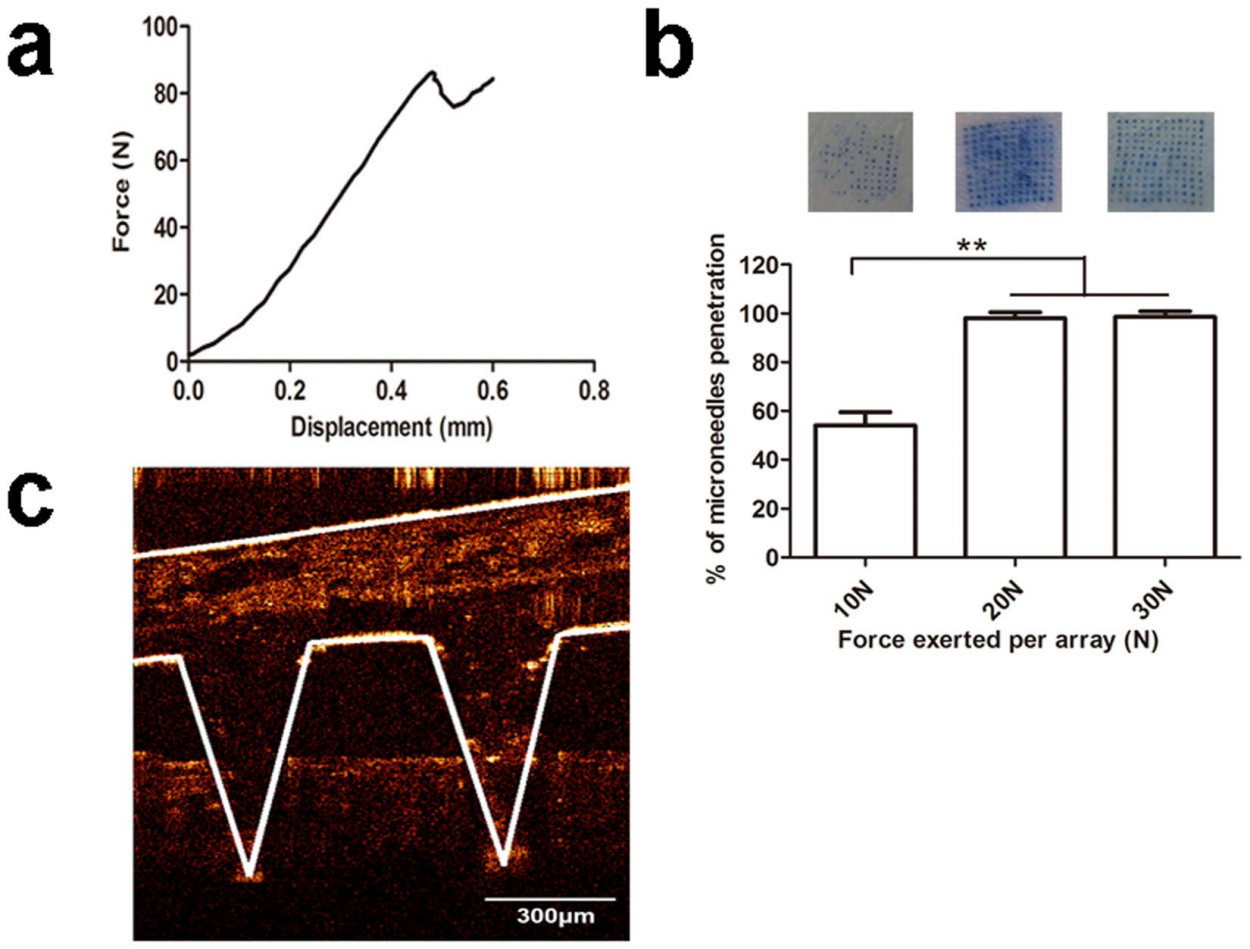
Mechanical strength of dissolving microneedles. (**a**) Fracture force determination of PEI/siRNA-loaded dissolving microneedles (n = 5). (**b**) Number of pinholes produced by different forces (10 N, 20 N, and 30 N) pressed on PEI/siRNA-loaded dissolving microneedles. Each value represented mean ± standard derivation (n = 3), **P < 0.01. (**c**) Optical coherence tomography (OCT) presented the depth of PEI/siRNA loaded microneedle puncturing into rat skin.

To obtain sufficient siRNA delivery into the skin, MNs should possess sufficient mechanical strength for puncturing the skin. The mechanical strength was characterized with the mechanical failure force. Figure 2a presented the force versus displacement curve of MNs. When the needle mechanically failed, the force decreased abruptly, and the value at this point was regarded as the needle mechanical failure force. The needle failure force of PEI/siRNA loaded MNs was ~86 N.

MNs punctured the skin by the pressing of external force. Skin pinhole created indicated that MNs penetrated the skin successfully. The pinhole number was calculated with the different external force of 10 N, 20 N, and 30 N, respectively. As shown in Fig. 2b, when the external force was 10 N, only 50% needles penetrated skin. However, when the external force increased to 20 or 30 N, almost 100% needles penetrated the skin. Therefore, 20 N was used to make the needles puncture the skin, and MNs maintained the shape during puncturing because the pressure was below the failure force of 86 N. Optical coherence tomography (OCT) was used to visualize and determine the depth of MNs penetration the skin *in situ* and real time. As shown in Fig. 2c, the depth of PEI/siRNA loaded MNs insertion into skin under the force of 20 N was ~330 μm, while the thickness of rat epidermis and whole skin were 32.1 ± 1.3 μm and 2.09 ± 0.07 mm, respectively^39^. Therefore, the results confirmed that the MNs presented enough mechanical strength to successfully puncture the rat skin, and deliver PEI/siRNA targeting to the epidermal basal layer and upper dermis, where melanoma cells were located.

### Dissolution of MNs in the skin

**Figure 3:**
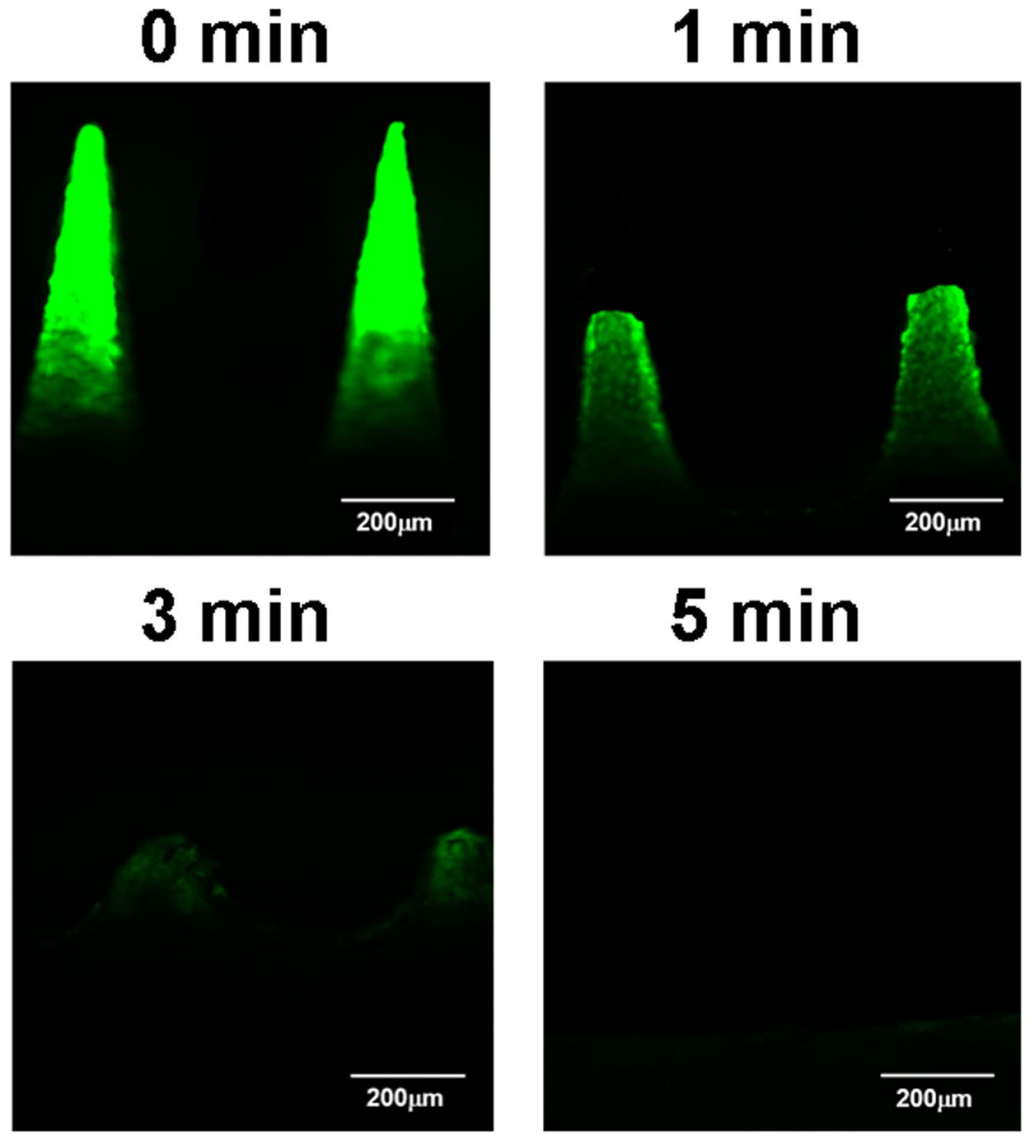
Micrograph of the residual PEI/FAM-siRNA loaded dissolving microneedles observed with confocal laser scanning microscope. The microneedles were pierced into the rat skin for maintaining 1 min, 3 min, or 5 min, and then removed.

To determine the dissolution profile of MNs in the skin, MNs loaded with PEI/FAM-siRNA were applied onto the skin with an external force of 20 N and removed after 1, 3, and 5 min administration. The residual MNs were observed with CLSM (Fig. 3). Figure 3 showed that the upper tip of MNs (~350 μm) dissolved in the skin after 1 min, and the microneedle body dissolved with time. After 5 min, the MNs fully dissolved in the skin, indicating that the MNs can dissolve in the skin upon contacting the interstitial fluid of viable epidermis/dermis within minutes and release siRNA loaded into the skin.

### *In vitro* cell assays

**Figure 4:**
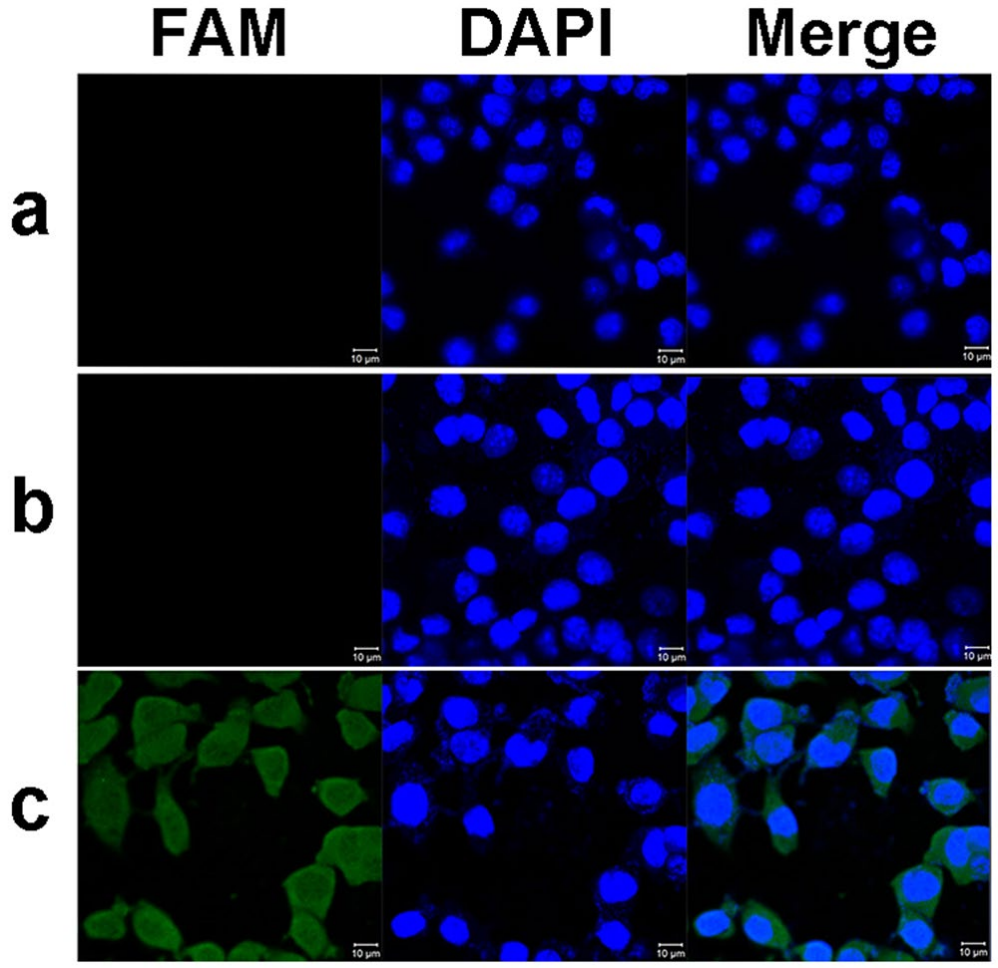
CLSM images of B16 cells uptake of PEI/FAM-siRNA complex. (**a**) Control; (**b**) Naked FAM-siRNA; (**c**) PEI/FAM-siRNA complex. Scale bar: 10 μm.

*Cellular uptake of PEI/FAM-siRNA*. Cellular uptake of PEI/FAM-siRNA with N/P ratio of 10 was assessed by CLSM and flow cytometry. CLSM was used to visualize the cellular uptake of FAM-siRNA by B16F10 melanoma cells. As shown in Fig. 4, B16F10 cells treated with PEI/FAM-siRNA presented strong green fluorescence around the cell nucleus in the cells, while no green fluorescence signals were observed in the cells incubated with naked FAM-siRNA. Hence, naked FAM-siRNA cannot be transferred into the cells owing to its negative charge, while PEI can effectively facilitate siRNA uptake by B16F10 cells.

**Figure 5:**
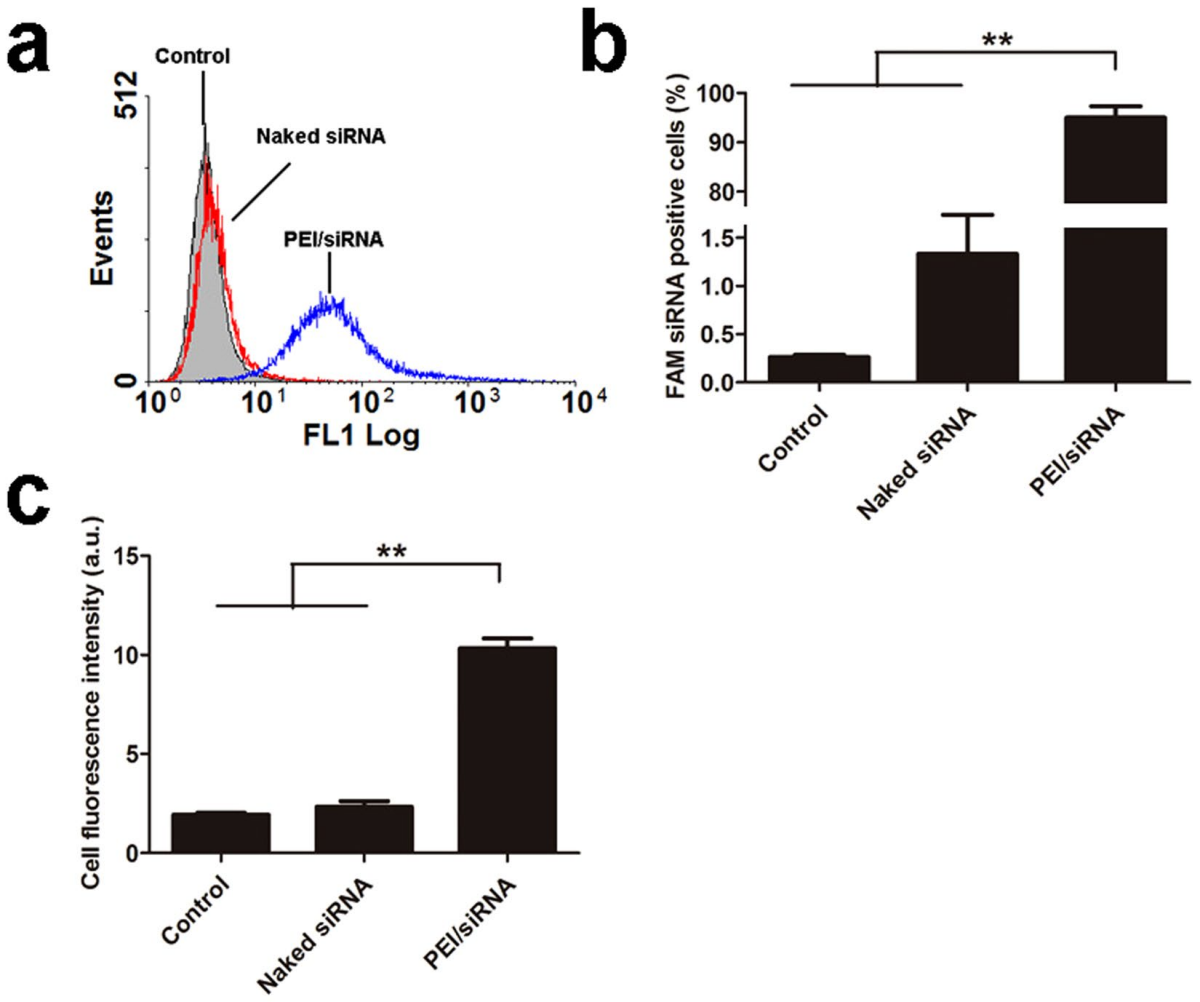
Cellular uptake of FAM-siRNA complex by B16 cells. (**a**) The image of characteristic flow cytometry histogram; (**b**) Percentage of FAM-siRNA positive B16 cells; (**c**) Average fluorescence intensity of B16 cells after 4 h incubation. Each value represented mean ± standard derivation (n = 3), **P < 0.01.

Flow cytometry was performed to quantitatively determine B16F10 cellular uptake of FAM-siRNA. Figure 5a showed the image of characteristic flow cytometry histogram. After incubation with PEI/FAM-siRNA for 4 h, the percentage of FAM positive B16F10 cells was ~100%, which was significantly higher than that of incubation with naked siRNA (~1%) (*P* < 0.01, Fig. 5b). In addition, the average fluorescence intensity of B16F10 cells incubated with PEI/FAM-siRNA was significantly higher than that of incubation with naked siRNA (*P* < 0.01, Fig. 5c).

In conclusion, results from CLSM and flow cytometry suggest that PEI can effectively enhance FAM-siRNA uptake into B16F10 cells.

### Gene silencing efficiency

**Figure 6:**
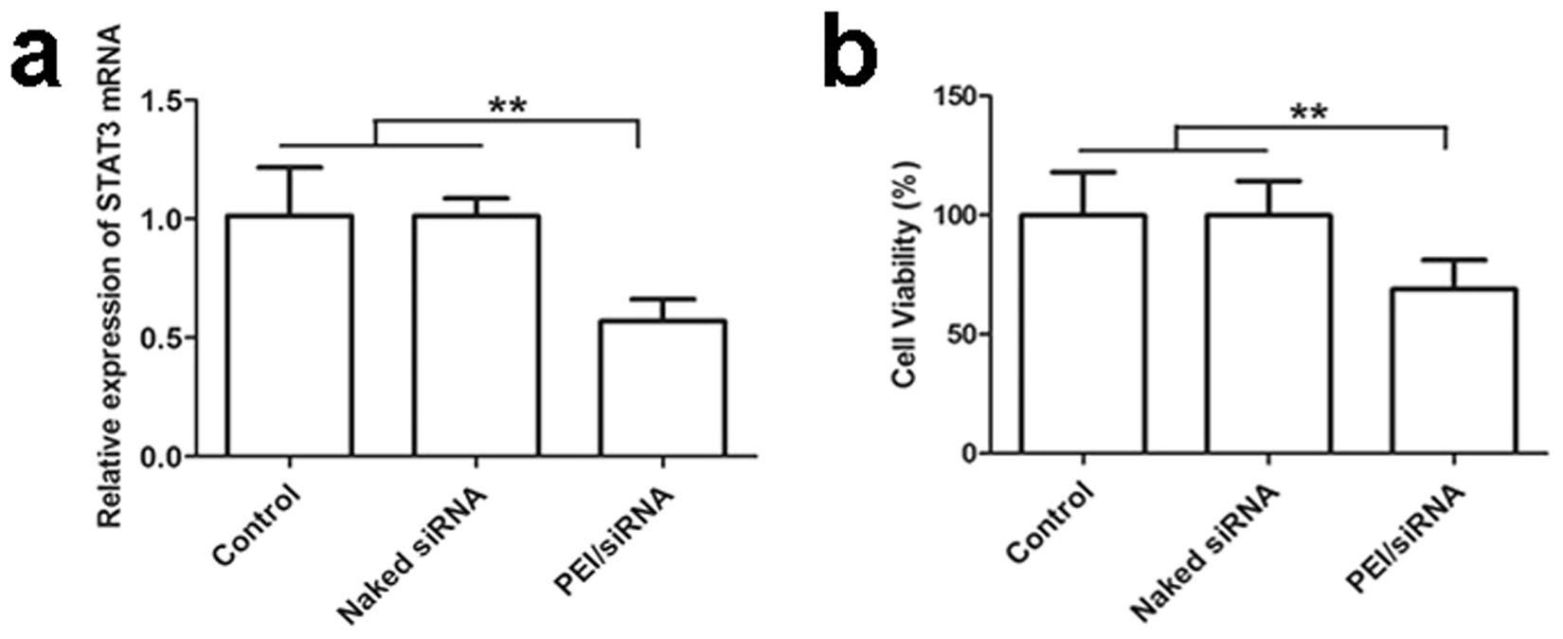
(**a**) *In vitro* STAT3 gene silencing. (**b**) Percentage of B16 cells viability was analyzed in comparison with the untreated control using MTT assay. Each value represented mean ± standard derivation (n = 3), **P < 0.01.

STAT3 plays an important role in many cellular processes including cell proliferation and apoptosis. It was hyperactive in malignant tumor cells, which makes it an attractive new target for cancer treatment. After being incubated with PEI/STAT3 siRNA of 100 nM, STAT3 gene silence of B16F10 cells was determined by RT-PCR. The STAT3 gene expression from the B16F10 cells treated with PEI/siRNA was significantly lower than that of the cells treated with naked STAT3 siRNA or untreated (*P* < *0*.01, Fig. 6a). The level of STAT3 mRNA of the B16F10 cells treated with PEI/STAT3 siRNA was down-regulated to 60.4%, while the level of STAT3 mRNA of the B16F10 cells treated with naked siRNA remained the similar to that of non-treated B16F10 cells. The results indicate that STAT3 siRNA can silence STAT3 gene of B16F10 cells, however, it must be first transferred into B16F10 cells via carrier, and PEI is an effective vehicle for transferring STAT3 siRNA into tumor cells.

### Anti-proliferation assays

To further assess the inhibition effect of STAT3 siRNA on B16F10 cells, 3- (4,5-dimethyl-2-thiazolyl)-2,5-diphenyl-2-H-tetrazolium bromide (MTT) was used to evaluate the cell viability. B16F10 cells proliferation was significantly inhibited when they were incubated with PEI/siRNA in comparison with the untreated group for 48 h (*P* < 0.01). As shown in Fig. 6b, B16F10 viability of PEI/siRNA treated reduced 33.6%, while that of naked STAT3 siRNA treated remained the same as that of non-treated B16F10 cells. The results further indicate that STAT3 siRNA delivered by PEI can effectively suppress B16F10 cell proliferation.

### Antitumor effect of STAT3 siRNA on melanoma in mice administrated by dissolving MNs and gene silencing *in vivo*

**Figure 7:**
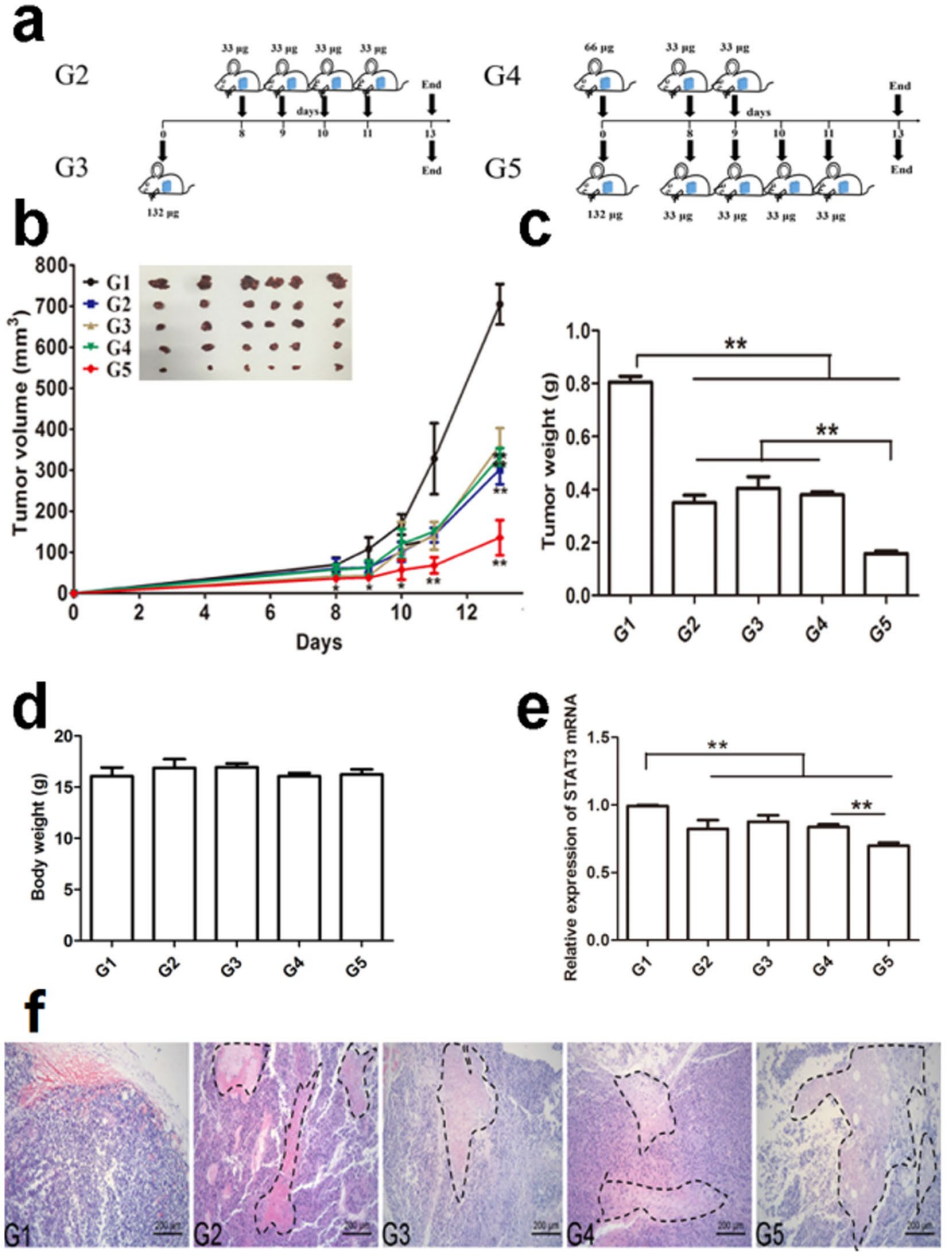
*In vivo* effect of PEI/STAT3 siRNA loaded with dissolving microneedles on melanoma. (**a**) Schematics of different administration protocols. (**b**) The average tumor volume versus time. The tumor volume of G2, G3, G4, or G5 was keeping significant smaller than that of G1 from the time point of day 9 to the termination of day 13 (^*^P < 0.05 at time point of day 9, and 10, and ^**^P < 0.01 at time point of day 11 and 13), and at time of day 8, the tumor volume of G3 or G5 significant smaller than that of G1 (*P* < 0.05). However, there was no significant difference between G2, G3, and G4 from the time point of day 9 to the termination of day 13. The tumor volume of G5 was further significantly smaller than that of G2, G3, or G4 (*P* < 0.01) (mean ± SD, n = 6; n = 6), (**c**) Tumor weight was compared at the end of experiment (mean ± SD, n = 6, **P < 0.01). (**d**) Body weight was determined at the end of experiment (mean ± SD, n = 6, **P < 0.01). (**e**) *In vivo* STAT3 gene silencing efficiency determined with RT-PCR (n = 3). (**f**) HE staining of representative tumor tissue from different groups. The tumor necrosis was outlined with black dotted lines.

To evaluate the antitumor effect of STAT3 siRNA on melanoma *in vivo*, we delivered PEI/siRNA topically to B16F10 melanoma tumor-bearing mice by dissolving MNs. Tumor-bearing mice were divided into 5 groups (group 1–5, G1-G5, 6 animals per group), and administration protocol was assigned with Fig. 7a except G1. G1 was the control group; the mice of G1 were not treated with any drug after B16F10 cells were implanted. For G2, G3, and G4, each mouse was administered with the same total dose of STAT3 siRNA (132 µg), whereas the total dose of G5 was double (264 µg). As shown in Fig. 7b, the tumor volume of G2, G3, G4, or G5 was keeping significant smaller than that of G1 from the time point of day 9 to the termination of day 13 (*P* < 0.05 at time point of day 9, and 10, and *P* < 0.01 at time point of day 11 and 13), and at time of day 8, the tumor volume of G3 or G5 significant smaller than that of G1 (*P* < 0.05). However, there was no significant difference between G2, G3, and G4 from the time point of day 9 to the termination of day 13. Interestingly, the tumor volume of G5 was further significantly smaller than that of G2, G3, or G4 (*P* < 0.01). The results reveal that different administration protocols with the same total dose exert the similar inhibition effects on tumor development, and the anti-tumor effect is dose-dependent.

At the time point of termination (day 13), the tumor was isolated and weighted. The tumor weight of G2, G3, or G4 was significantly lower than that of G1(*P* < 0.01, Fig. 7c), and about half of that of G1. The tumor weight of G5 was further significantly lower than that of G2, G3, or G4 (*P* < 0.01), and about one fifth of that of G1. Based on the results from tumor volume and weight, it can be concluded that the topical delivery of PEI/siRNA via dissolving MNs can inhibit the tumor growth of melanoma, and the inhibition effect is dose-dependent.

There was no significant difference of the body weight of tumor-bearing mice after different treatment from G2 to G5 in comparison with the untreated group of G1 (Fig. 7d); the therapy with PEI/siRNA of different administration protocols or doses for melanoma did not cause body weight loss. The results might be attributed to the targeting delivery of STAT3 siRNA to melanoma via dissolving MNs and the selective effect of STAT3 siRNA on STAT3 gene of melanoma cells.

After the termination of the treatment experiment, tumor tissues from different groups were isolated and total RNA was extracted. RT-PCR was used to measure STAT3 mRNA expression. As shown in Fig. 7e, the relative mRNA expression level of G2, G3, and G4 was down-regulated to 82%, 87%, and 83% respectively in comparison with the untreated group (*P* < 0.01), and there was no significant difference between G2, G3, and G4. However, the relative mRNA expression level of G5 was significantly lower than that of G2, G3, or G4 (*P* < 0.01), and it was reduced to 70%. Consistent with the inhibition effects of PEI/siRNA on tumor volume and weight, the gene silencing effects of PEI/siRNA on tumor tissue was confirmed *in vivo*, and the silencing effect was also dose-dependent. The gene silencing effects of PEI/siRNA on STAT3 mRNA expression *in vivo* was the antitumor molecular mechanism of STAT3 siRNA against melanoma.

Based on the histological analysis (Fig. 7f), tumor of G1 was rich in blood vessels, and the tumor cells were pleomorphic with heavy density. However, the tumor in different treated groups presented necrosis with a percentage of ~20% in G2-G4, and ~40% in G5. The tumor necrosis area was outlined with dotted line. The histological results provide further evidence that the topical delivery of PEI/siRNA via dissolving MNs can inhibit melanoma *in vivo*, and the inhibition effect is dose-dependent.

## Discussion

In recent years, gene silencing technology has developed rapidly and become a potential therapy for human illnesses. Among which, siRNA therapy is the most hopeful and has received widespread attention^40–42^. Currently, new siRNA-based medicines and siRNA therapy have been developed in clinical trials^41,43^. Malignant tumors are genetic diseases involved a line of cumulative epigenetic and mutational changes which cause uncontrollable cell metastasis, differentiation and proliferation^44,45^. The major advantage of siRNA technology is specificity by the principle of Watson-Crick base pairing to discriminate target gene versus non-target gene^46^. A series of preclinical researches have indicated that gene silencing could suppress tumor development involving tumor cell apoptosis, proliferation, angiogenesis, metastasis, immune escape, and chemo-resistance^47–49^. STAT3 is hyperactive in melanoma, its abnormal activation promotes tumor cell proliferation, suppresses apoptosis, and induces invasion and metastasis; therefore, silencing STAT3 gene by siRNA is a promising strategy for treating melanoma^50,51^. However, applying siRNA gene therapy for skin melanoma, which initiates in the basal layer of epidermis and upper dermis *in vivo*, faces two obstacles: delivery and transfection. While intradermal delivery technology breaches the barrier function of SC, further carrier is required to facilitate cellular uptake of siRNA. The goal of our study was to design a dissolving microneedle system to deliver STAT3 siRNA into the skin in a targeted manner to breach the stratum corneum and directly reach the site of basal layer of epidermis and upper dermis, and PEI was used as the carrier to increase the transfection efficiency. It is evident that siRNA can form tight complex with the cationic PEI via attractive electrostatic interactions and be internalized by cells with the help of cationic complex^50,52^. PEI /siRNA of our current study can effectively facilitate siRNA uptake by B16F10 cells, the percentage of FAM positive B16F10 cells approached ~100% after incubation with PEI/FAM-siRNA for 4 h, which was significantly higher than that of incubation with naked siRNA (~1%), and the complex down-regulated STAT3 mRNA to 60.4%, B16F10 viability of PEI/STAT3 siRNA treated decreased by 33.6%, while the gene expression and cell viability of B16F10 cells treated with naked STAT3 siRNA almost remained the similar to those of non-treated B16F10 cells. These results were consistent with previous findings that naked STAT3 siRNA presented no significant inhibition on B16F10 cells growth^51^. STAT3 siRNA can silence STAT3 gene of B16F10 cells; however, it must be first transferred into B16F10 cells via carrier due to its own poor cellular permeability, and PEI is an effective vehicle for transferring STAT3 siRNA into B16F10 cells. Development of efficient and safe carrier to transfer STAT3 siRNA into cells will be critical for its clinical application, which will be the subject of our further research.

MNs, a minimally-invasive, patient-friendly, and self-administered approach, can overcome SC barrier and deliver the payload to viable epidermis and dermis, and modulation of MNs geometry and formulations can result in controlled drug deposition within targeted skin layers^29^. MNs presented a promise for intradermal delivery of macromolecules, and coated steel MNs^53^, hollow MNs^37,54^, and solid MNs^38^ have been reported to deliver siRNA into the skin for treating skin disorder caused by aberrant gene expression. Compared to the other MNs (solid, coated, or hollow MNs), dissolving MNs can dissolve in the skin interstitial fluid of viable epidermis/dermis within minutes and release the drug payload, avoiding biohazardous sharp waste in the skin and reducing any risk of infection transmission^55,56^.

The study is the first to explore the potential of dissolving MNs for delivering STAT3 siRNA into skin *in vivo* and subsequent anti-melanoma and gene silencing efficacy. The designed dissolving MNs based on dextran, HA, and PVP with a ratio of 4:1:1 (w/w/w) possessed mechanical strength of ~86 N, and almost all the microneedle arrays in a given external force of 20 N punctured the skin and remained in shape in the skin. The depth of insertion was ~330 μm, which ensured delivering the siRNA loaded into the epidermal basal layer and upper dermis, where melanoma cells were located. However, MNs cannot penetrate the skin to their full length (~650μm), which is consistent with previous reports^56,57^. Therefore, we optimized the drug loading method to make siRNA preferentially located toward the upper tip of MNs, thereby enabling complete siRNA delivery even with incomplete microneedle penetration. The CLSM images before and after administration of MNs showed that MNs fully dissolved within 5 min after puncturing the skin. We therefore expect that the MNs can dissolve in the skin upon contacting the interstitial fluid of viable epidermis/dermis within minutes and release and deposit the siRNA loaded into the skin.

As expected, the dissolving microneedle effectively delivered PEI/siRNA topically to B16F10 melanoma tumor-bearing mice and exerted the tumor inhibition efficacy of siRNA, and the anti-tumor effect was dose-dependent on siRNA. After being treated with total dose of 264 µg of STAT3 siRNA, the tumor volume and weight decreased by ~80% compared with the untreated animal (135.57 ± 43.01 mm^3^ *vs* 704.77 ± 49.36 mm^3^, 0.16 ± 0.02 g *vs* 0.80 ± 0.05 g), while being treated with total dose of 132 µg of STAT3 siRNA, the tumor volume and weight decreased by ~50% (361.34 ± 41.47 mm^3^ *vs* 704.77 ± 49.36mm^3^, 0.40 ± 0.07 g *vs* 0.80 ± 0.05 g). However, different administration protocols with the same total dose of STAT3 siRNA exert the similar inhibition effects on tumor development. The tumor necrosis observed from histological analysis further confirmed the anti-tumor results. Moreover, there was no significant difference of the body weight of tumor-bearing mice with different treatment protocols and doses, which might be attributed to the targeting delivery of STAT3 siRNA to melanoma via dissolving MNs and the selective effect of STAT3 siRNA on STAT3 gene of melanoma cells. Consistent with *in vitro*, PEI/siRNA showed an effective down regulation of STAT3 mRNA expression *in vivo*, which was the antitumor molecular mechanism of STAT3 siRNA against melanoma. We demonstrated for the first time that dissolving microneedle is an effective and safe strategy for targeting delivery of STAT3 siRNA via skin to melanoma cells and exerts specific anti-melanoma effect.

## Materials and Methods

### Animals

Female C57BL/6 mice (6–8 weeks) and Sprague Dawley (SD) rats (200–220 g) were obtained from Experimental Animal Center of Guangzhou University of Chinese traditional medicine (License number: SCXK (YUE) 2013–0034). All protocols for animals were in accordance with the Institutional Animal Care and Use of Sun Yat-sen University.

### Ethics statement

All experimental protocols were approved by the Animal Experimentation Ethics Committee of Sun Yat-sen University.

### Cell culture

Mouse melanoma cell line B16F10 was purchased from American Type Culture Collection (ATCC, VA, USA) and incubated in RPMI-1640 medium supplemented with 10% FBS at 37 °C with 5% CO_2_.

### SiRNAs

The siRNA targeting murine STAT3 mRNA, negative control siRNA, FAM-labeled siRNA, and STAT3 primers were synthesized by Gene Pharm (Shanghai, China). β-actin primers were bought from Sangon Biotech (Shanghai, China). The sequence is: STAT3 siRNA: sense: 5′-GGACGACUUUGAUUUCAACtt-3′; antisense: 5′-GUUGAAAUCAAAGUCGUCCtg-3′. Negative control siRNA: sense:5′-UUCUCCGAACGUGUCACGUTT-3′; antisense: 5′-ACGUGACACGUUCGGAGAATT-3′. β-actin primer forward: 5′-CCAACCGCGAGAAGATGA-3′; reverse: 5′-CCAGAGGCGTACAGGGATAG-3′.

### Preparation of PEI/siRNA complex

siRNA was added to the same volume of PEI solution at the N/P ratio of 10:1, and the mixture was incubated at room temperature for 30 min to obtain PEI/siRNA complex. The diameter and potential of the complex determined with Dynamic Light Scattering (Malvern Instruments NanoZS90, Worcestershire, UK) were 135.1 ± 5.2 nm and 34.6 ± 2.2 mV, respectively.

### Fabrication of dissolving MNs

A stainless pyramidal mould of 144 (12 × 12) MNs with height of 650 μm and base of 300 μm was used as the master mould. The inverse mould was prepared by casting polydimethylsiloxane (PDMS) over the master mould and degassed in a vacuum desiccator for 1 h followed by curing in the 95 °C oven for 30 min to solidify the polymer. Then the PDMS mould was obtained after peeling off the master mould and repeatedly used to fabricate dissolving MNs.

A two-step moulding process was applied to prepare drug-loaded dissolving MNs. Firstly, a mixture of dextran 40, PVP 17, and HA at a weight ratio of 4:1:1 was dissolved in ultrapure water, and then Rh-B, PEI/STAT3 siRNA, or PEI/FAM-siRNA was added and mixed uniformly to obtain drug-loaded needle solution. Fifty milligrams drug-loaded needle solution was dipped into PDMS mould, and the mould was degassed under vacuum for 10 min and then centrifuged at 4000 rpm for 20 min so that the needle solution filled the PDMS mould microcavities completely, obtaining the needle tip layer. Secondly, a mixture of dextran 40, PVP 17, and HA at a weight ratio of 3:3:1 was dissolved in ultrapure water to obtain the base solution. One hundred fifty milligrams of base solution without drug was casted onto the tip layer surface, then centrifuged at 4000 rpm for 10 min and dried for 24 h at 4 °C. Eventually, the drug-loaded dissolving MNs was obtained by peeling off from PDMS mould. The dissolving MNs loaded with Rh-B, PEI/STAT3 siRNA, or PEI/FAM-siRNA were respectively characterized with digital camera (Canon, Japan), scanning electron microscope (SEM, JSM-6330F, Jeol, Tokyo, Japan), and confocal laser scanning microscope (CLSM, Zessi LSM 710, Germany). Rh-B, PEI/STAT3 siRNA, or PEI/FAM-siRNA MNs were stored in 4 °C and away from light.

### Mechanical strength and skin insertion studies

A TA.XT plus texture analyzer (Stable Micro Systems, Surrey, UK) was used to determine the mechanical properties of PEI/siRNA loaded MNs under an axiload. Stress versus strain curves were generated with force and displacement. The test station pressed MNs against a rigid metal surface at a rate of 1.1 mm/s. The texture analyzer recorded the force until a preset displacement of 600 μm was reached.

To measure the insertion efficiency of dissolving MNs, excised SD rat skin was used as skin model. The back skin was shaved, excised, subcutaneous fat removed, and cleaned with PBS and ethanol; then the skin was stretched on Styrofoam and fixed with pins. The microneedle patch was attached to the removable cylindrical probe of texture analyzer with double sides adhesive tape. The microneedle patch following the probe inserted the skin at a rate of 1.1 mm/s with different forces of 10 N, 20 N, and 30 N, respectively, and retained in the skin for 60 s. After the MNs were removed from the skin, trypan blue solution (0.5%, w/v) was added onto the punctured skin and stayed for 10 min, trypan blue would penetrate through the pinholes. After washing off the trypan blue solution, the stained pinholes in the skin were calculated.

The depth of MNs insertion into the skin was measured by optical coherence tomography (OCT, TEK SQRAY HSO-2000, China) . The rat skin was treated as above described and placed on a PDMS substrate for support. MNs inserted the skin with a force of 20 N by thumb pressing. Then the depth of insertion was observed with OCT in real time. The OCT parameters were set as follows: low-coherence light with power of 5 mW, full width at half maximum (FWHM) of 45 nm, laser center wavelength at 830 nm, and scanning rate at 18 kHz B-scans. Matlab Version 7.14 (MathWorks Inc., Massachusetts, USA) was used to image the skin layers and MNs.

### Dissolution of microneedles

PEI/FAM-siRNA loaded MNs inserted the rat skin for 0, 1, 3 or 5 min respectively and then were removed. CLSM was used to observe the residual length of MNs.

### *In vitro* cell assays

*CLSM*. CLSM was used to visualize the uptake of FAM-labeled siRNA by B16F10 cells. B16F10 cells of 1 × 10^5^ were cultured in dishes. PEI/FAM-siRNA or naked FAM-siRNA were added at a final siRNA concentration of 100 nM and incubated with cells for 4 h. Afterwards, the cells were washed with PBS for four times and fixed with 4% paraformaldehyde for 30 min. DAPI was used for dyeing cell nucleus. Finally, the samples were visualized under CLSM. He Ne laser was used to excite FAM at 488 nm.

#### Flow cytometry

For quantitative evaluation of transfection efficiency, the cellular uptake of FAM-siRNA was measured by flow cytometry. After B16F10 cells was transfected with PEI/FAM-siRNA or naked FAM-siRNA at a final siRNA concentration of 100 nM as above described, the percentage of FAM positive B16F10 cells and average intensity of fluorescence were measured with flow cytometry.

#### Real-time PCR

RT-PCR was performed to confirm the silencing efficiency of STAT3 siRNA on STAT3 gene of B16F10 cells. After B16F10 cells were transfected for 4 h with naked STAT3 siRNA and PEI/STAT3 siRNA respectively at a final siRNA concentration of 100 nM. The residual uninternalized complex was removed with RPMI 1640 and the cells were continued to incubate for 24 h. Afterwards, the cells were washed with PBS for three times, and trizol was added to extract total RNA following the instruction protocol. Total RNA of 500 ng as loading control was treated with PrimeScript^TM^ RT reagent kit with gDNA eraser (TAKARA BIO INC, Japan). Reverse Transcription System (Promega, Madison, USA) was used to synthesize cDNA, and SYBR Green was employed to real-time PCR. LightCycler480 II (Agilent Technologies, USA) was used to measure the threshold cycle number (Ct) of STAT3 and β-actin. STAT3 and β-actin mRNA was calculated by 2^−ΔΔ Ct^ relative-quantitative method.

#### Anti-proliferation assays

Cell growth was measured with MTT cell proliferation assay. Briefly, 5 × 10^3^ B16F10 cells were cultured in 96-well plates overnight, then the cells were transfected with PEI, PEI/siRNA or naked STAT3 siRNA (100 nM siRNA per well), respectively. After 4 h transfection, the uninternalized complexes was removed with RPMI 1640 and the cells were continued to incubate for 48 h. Cell proliferation activity was measured by adding 20 μL of MTT per well. After cultivation for 4 h, 150 μL of DMSO was added to each well to dissolve formazan crystals. The optical density (OD) value was detected at 490 nm using FlexStation III Multi-Mode Microplate Reader (Molecular Devices, California, USA).

### Antitumor evaluation

#### *In vivo* melanoma model

Female C57Bl/6 were weighted and randomly divided into different groups (6 mice per group). Each mouse was subcutaneously injected with 1 × 10^6^ B16F10 cells in the left flank, and melanoma reached 50–60 mm^3^ on day 8 after inoculation based on our preliminary experiment. After inoculation, the mice were treated as follows: 1) Group 1 (G1) was the tumor model control group without administration; 2) Group 2 (G2), each mouse was administered with PEI/siRNA of 33 μg per day via MNs on day 8 after inoculation and for 4 consecutive days (Fig. 7G2); 3) Group 3 (G3), each mouse was administered with PEI/siRNA of 132 μg via MNs once the tumor cells were inoculated (Fig. 7G3); 4) Group 4 (G4), each mouse was administered with PEI/siRNA of 66 μg via MNs once the tumor cells were inoculated, and then administered with PEI/siRNA of 33 μg per day via MNs on day 8 and 9 after inoculation (Fig. 7G4); 5) Group 5 (G5), each mouse was administered with PEI/siRNA of 132 μg via MNs once the tumor cells were inoculated, and then administered with PEI/siRNA of 33 μg per day via MNs on day 8 after inoculation and for 4 consecutive days (Fig. 7G5). Therefore, the total dose of each mouse was the same of 132 μg for G2-G4, and double for G5 of 264 μg.

Eight days after inoculation tumor cells, tumor size of each mouse was detected with digital caliper over time. The tumor volume was calculated by the following formula:

$${\rm{tumor}}\,{\rm{volume}}\,({{\rm{mm}}}^{{\rm{3}}})={\rm{\pi }}\times {\rm{length}}\times {{\rm{width}}}^{{\rm{2}}}/{\rm{6}}$$

Moreover, mouse body weight was monitored daily to evaluate the potential toxicities. At the time point of day 13, the mice were euthanized. Tumors were immediately isolated, washed with PBS to remove blood, dried with tissue paper, and weighted. The tumor tissue was sectioned, fixed in 4% paraformaldehyde, dehydrated, embedded in paraffin. Slice of 3 μm tumor tissue was stained with standard HE procedure. Slices were observed with optical microscope (DM5000B, Leica, Germany) for histological analysis.

#### Gene silencing *in vivo*

To assess gene silencing effects *in vivo*, the clean tumor tissue was treated with trizol solution and homogenized to form uniform cell suspension by ultrasonic cell disruptor (VCX 130PB, Sonics & Materials Inc., Newtown, USA). Subsequently, total RNA was extracted and analyzed with RT-PCR as mentioned *in vitro*.

### Statistical analysis

All measurements were performed at least in triplicate. The data presented as mean ± standard deviation (SD). The statistical difference was compared with one-way analysis of variance (ANOVA) followed by Least Significant Difference (LSD) as post hoc analysis (SPSS version 19.0; SPSS Inc., Chicago, IL, USA). Statistical significance was set at P < 0.05.

### Data Availability

The datasets generated and/or analyzed during the current study are available from the corresponding author on reasonable request.

## Electronic supplementary material

Supplementary information
