# Supplementary material for: Intradermal delivery of STAT3 siRNA to treat melanoma via dissolving microneedles
Source: Sci Rep. 2018 Jan 18;8:1117. doi: 10.1038/s41598-018-19463-2 (PMC5773564; doi:10.1038/s41598-018-19463-2)
Supplement: Supplementary file 1 — Supplementary information [file 41598_2018_19463_MOESM1_ESM.pdf]

Supplementary Information

Intradermal delivery of STAT3 siRNA to treat melanoma via dissolving microneedles

Jingtong Pan, Wenyi Ruan, Mengyao Qin, Yueming Long, Tao Wan, Kaiyue Yu, Yuanhao Zhai, Chuanbin Wu & Yuehong Xu*

Supplementary 1

The TUNEL staining of tumor tissue to further illustrate the mechanisms of PEI/siRNA on tumor, and the results of TUNEL staining was shown as follows (Figure s1). Based on the histological analysis, tumor of control group (G1, without siRNA treatment) has complete cell form and TUNEL-positive cells (brown cell) were hardly to be observed. However, the tumor treated with different formulation of STAT3 siRNA (group 2-5, G2-G5) presented TUNEL-positive with a percentage of ~30% in G2-G4, and ~50% in G5. The histological results provide further evidence that the topical delivery of PEI/ siRNA via dissolving MNs can promote the apoptosis of melanoma in vivo, and the promotion effect is dose-dependent.

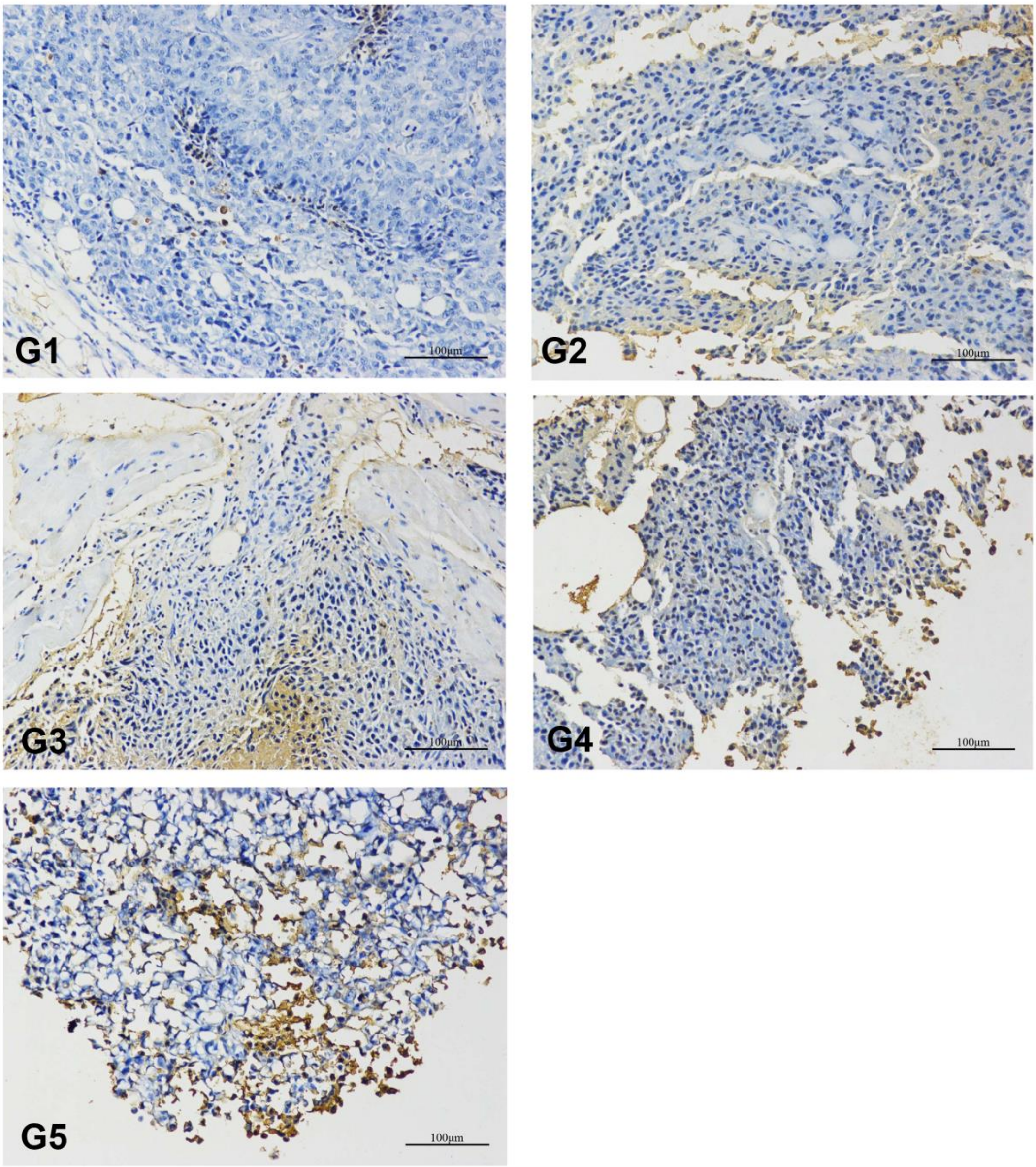

Figure s1 Tumor cross-sections stained with TUNEL technique using TUNEL kit (Roche, Switzerland). Scale bar: 100 µm.

Supplementary 2

The experiment of in vivo imaging with the aim to observe the distribution of siRNA delivered with MNs. Cy 5.5-labeled siRNA was incubated with PEI to prepare PEI/ Cy 5.5-siRNA complex, and then PEI/ Cy 5.5-siRNA complex was loaded into microneedle. The microneedle patch loaded with PEI/ Cy 5.5-siRNA complex was locally administered onto the tumor site of xenograft model of C57Bl/6 mouse for 5 min and then removed. The skin of tumor site was cleaned, and the mouse was visualized with the NightOWL II LB983 in vivo imaging system (BERTHOLD, Germany). Figure s2 presented the in vivo imaging of the mouse. As shown in Figure s2, it was observed that the tumor site presented the strongest fluorescence intensity, indicating that PEI/Cy 5.5-siRNA complex was locally delivered into the tumor via microneedle and accumulated in the tumor, and the siRNA would be transferred into melanocytes assisted by PEI.

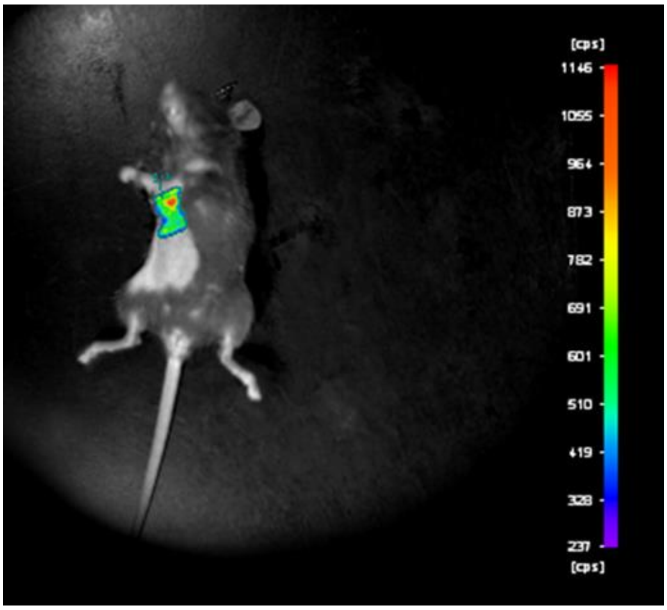

Figure s2. Fluorescence intensity of Cy5.5-labeled siRNA in C57Bl/6 mice bearing xenografted B16F10 tumor after treated with PEI/ Cy5.5-siRNA MNs.

Supplementary 3

The results of STAT3 siRNA transferred by lipofectamine^{TM} 2000 (Invitrogen, USA) and measured the gene silencing efficiency by RT-PCR were supplemented. Figure s3 shows the results of STAT3 siRNA transferred by lipofectamine^{TM} 2000 and gene silencing efficiency compared with naked siRNA or PEI/siRNA. The gene silencing efficiency of Lipo/siRNA complexes is slightly higher than that of PEI/siRNA complexes. However, we chose PEI as the carrier based on the cost-effective consideration.

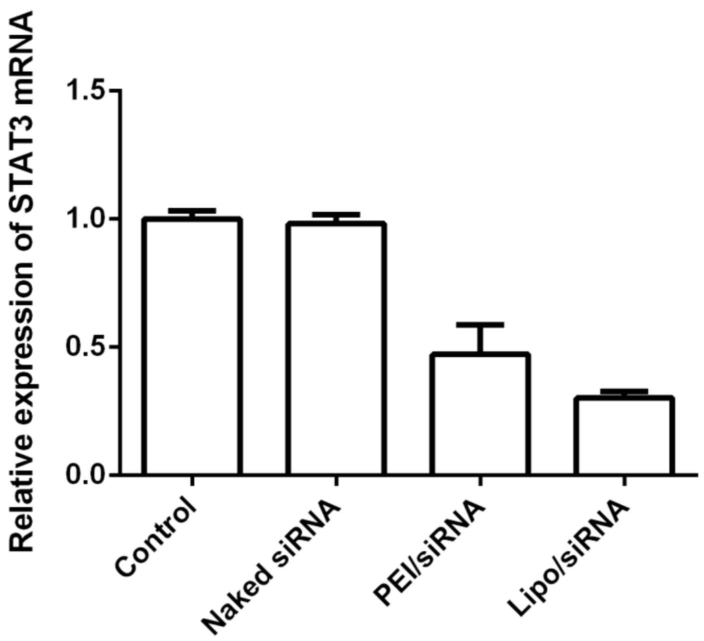

Figure s3. In vitro STAT3 gene silencing of B16F10 cells transferred with different STAT3 siRNA formulations.
